# A new innovative real-time tracking method for flying insects applicable under natural conditions

**DOI:** 10.1186/s40850-021-00097-3

**Published:** 2021-12-23

**Authors:** Thomas Walter, Jacqueline Degen, Keram Pfeiffer, Anna Stöckl, Sergio Montenegro, Tobias Degen

**Affiliations:** 1 Department of Computer Science, Aerospace Information Technology, Emil-Fischer-Str. 70, Würzburg, 97074 Germany; 2 Department of Zoology II, Behavioral Physiology and Sociobiology, Am Hubland, Würzburg, 97074 Germany; 3 Department of Zoology III, Theoretical Evolutionary Ecology Group, Emil-Fischer-Str 32, Würzburg, 97074 Germany

**Keywords:** Natural environment, Insect tracking, Real-time, Movement ecology, Heterogeneous background

## Abstract

**Background:**

Sixty percent of all species are insects, yet despite global efforts to monitor animal movement patterns, insects are continuously underrepresented. This striking difference between species richness and the number of species monitored is not due to a lack of interest but rather to the lack of technical solutions. Often the accuracy and speed of established tracking methods is not high enough to record behavior and react to it experimentally in real-time, which applies in particular to small flying animals.

**Results:**

Our new method of real-time tracking relates to frequencies of solar radiation which are almost completely absorbed by traveling through the atmosphere. For tracking, photoluminescent tags with a peak emission (1400 nm), which lays in such a region of strong absorption through the atmosphere, were attached to the animals. The photoluminescent properties of passivated lead sulphide quantum dots were responsible for the emission of light by the tags and provide a superb signal-to noise ratio. We developed prototype markers with a weight of 12.5 mg and a diameter of 5 mm. Furthermore, we developed a short wave infrared detection system which can record and determine the position of an animal in a heterogeneous environment with a delay smaller than 10 ms. With this method we were able to track tagged bumblebees as well as hawk moths in a flight arena that was placed outside on a natural meadow.

**Conclusion:**

Our new method eliminates the necessity of a constant or predictable environment for many experimental setups. Furthermore, we postulate that the developed matrix-detector mounted to a multicopter will enable tracking of small flying insects, over medium range distances (>1000 m) in the near future because: a) the matrix-detector equipped with an 70 mm interchangeable lens weighs less than 380 g, b) it evaluates the position of an animal in real-time and c) it can directly control and communicate with electronic devices.

## Background

Recording and analysing animal movement patterns is invaluable not only for biological research, but also for evaluating and assessing ecological and economic issues [1–6]. To avoid misunderstandings, we would like to frame our definition of tracking as follows: Waypoints, i. e. coordinates indicating the position of the object to be observed can be acquired anywhere along its route or line of travel, apart from systemic exceptions (e.g. obstacles like trees for harmonic radar).

There are various methods to track animals. In general, a tracking method must meet different requirements to be used in natural or artificial environments. In contrast to an artificial environment, most parameters are not controllable in a natural environment (e. g. the background) and recording of more than a few waypoints of a route or line of travel is still challenging for small animals (<100 g) [6–8]. In the field, many different techniques utilizing different signals, e. g. electromagnetic- and acoustic waves, are used to track individuals. However, to the best of our knowledge, methods for tracking individual insects are limited to electromagnetic radiation. Thus, the focus of the manuscript lies exclusively on methods that use electromagnetic radiation.

As mentioned before it is still difficult to record movement patterns of small animals like honey bees — even if they are tagged [5, 6]. Honey bees are considered to be strong flyers capable of coping with payloads representing 20% of their body mass without any indication of an altered flight performance [9]. For flying animals in general, only attached items that weigh less than 5% of the body are assumed not to affect flight performance — "5% rule“ — even if this assumption is not based on a very large data base [7, 10, 11]. Honey bees and butterflies (*Aglais urticae*, *Melitaea cinxia*, *Agrotis segetum*) mark the bottom end of the technical possible range of trackable flying animals[12]. So far smaller flying insects can only be recorded in the field as assemblages [6, 13, 14].

The high demand for technical solutions to track small insects has led to technical progress. Thus, transmitters and transponders (active, semi-active, and passive), with which animals were equipped for tracking, became smaller and lighter over time. [7, 15–17]. In general, passive transponders (without their own power source) are much lighter than active and semi-active transponders and transmitters (which have their own power source).

The newest generation of battery powered active radio frequency (RF) identification tag weigh approximately 95 mg and reach an operational distance of about 1 km with a 10 cm whip-antenna [16]. However, these transmitters have not been tested for their suitability to track animals yet. Although this approach is very promising, the length of the antenna is likely to be problematic [6, 12]. It might be reasonable to increase the frequency if technically possible, even if the lifespan — currently 16 days — of the tag might be reduced due to a higher power consumption and a higher susceptibility to interference. Another drawback of this new approach might be that it is based on complementary metal-oxide-semiconductor (CMOS) technology where the unit price extremely depends on the number of pieces produced. Finally, although 95 mg is a huge step forward as it represents a weight reduction of over 50% compared to other systems of this kind, it still adds about 80% of the weight of a honeybee. Such high payloads most likely affect energy consumption and flight performance [12, 14, 18–20].

A new unique and very promising approach utilizes a batteryless active radio frequency (RF) transmitter (which weighs 30 mg to 80 mg) with a small stationary receiver. Instead of having a battery it harvests the required energy (for the transmitter) via an piezoelectric harvester. The authors showed that their system works with an operational distance of about 10 m. Furthermore, they autonomously tracked a hand held battery equipped transmitter over a distance of 50 m with a drone. Thus, in near future it could be the first non-stationary tracking device mounted on a multicopter with which tracking of insects would be possible [17]. According to their own statement the lightest tags (30 mg) are not easy to produce, which results in a high reject rate. It is possible that this method will soon be superior to radar technology in many respects, but at least in one respect it is not - the transmitters weigh at least twice as much as conventional radar tags [5, 14, 21].

To our knowledge all devices used in the field for passive tags are stationary ones with a small detection range of only a few centimetres up to some meters. The only exception is the harmonic radar system that can reach distances up to 1 km [6, 21–23]. Harmonic radar tags require an antenna which is between 12 mm to 16 mm long. Despite the fact that, due to the frequency, the antennas used are relatively short, various reviews of tracking methods point out that this is still a disadvantage of the method which might influence the behaviour of the animals under investigation [6, 12, 24].

Due to the utilized wavelength, harmonic radar tracking only works in a flat terrain without obstacles like trees or bushes [5, 25]. Another drawback is the low resolution in space (±2.5 m) and time (≥3 sec). Thus, it is not possible to distinguish between individuals if they meet or fly past each other within a small distance [6, 25]. However, for bumblebees Riley [26] found a mean flight speed of 7.1 ms^-1^ — in windless conditions. This means that a straight flight starting at the center of the largest possible area to be observed with a harmonic radar (radius<1000 m) is covered in less than 1.2 min. Thus, all prior stationary devices — even the harmonic radar — are just a peek inside the movement patterns of flying animals if they are not central place foragers[7].

There are two ways to address these shortcomings: a) by using many stationary devices or b) by tracking individuals with a non-stationary device. Since movement in space takes place in at least two dimensions — under the very strong simplification that the flight altitude is negligible, the number of stationary devices required for surveillance of a certain study site increases proportionally to its size. Therefore, the use of passive tags with a short detection range — not to mention the costs — is logistically hardly manageable, simply due to the above named relation. Thus, even if radio frequency identification (RFID) tags with a range of about 1.5 m and a weight of about 10 mg will be available in the near future [15], they will not be suitable for experiments comparable to those with harmonic radar. Consequently, in the foreseeable future, only b) remains as a feasible solution. One can easily imagine that if the mobile unit (i. e. the receiver or transceiver) would be airworthy — e. g. by mounting it to a drone, this would be a great solution. But it should be noted that the more thrust (i. e. the heavier the flying object) is required, the greater the downwash and therefore the distance to the observed object required for keeping it under a defined level of influence. On the transceiver/receiver’s side, there seems to be only one clear solution, to make it mobile.

A yet unanswered question for tracking is whether the size or the radio frequency band of the transponder/transmitter is more important. In general, when using radio frequencies for communication, it should be noted that the frequency used has a decisive influence on the susceptibility of the system to interference (i.e. disruptive effects of the environment)[6, 12, 27–29]. The susceptibility to interference from water and reflections correlates positively with the frequency used [27–29]. This is particularly important when tracking animals in the wild, as the signal has to travel through the (natural) environment.

However, since the optimal antenna length is proportional to the wavelength, lower frequencies require longer antennas which affects tag (i.e. transmitter or transponder) size [27] — consequently, miniaturisation has its price. Interestingly, the harmonic radar uses the lightest tags (with super high frequency (SHF) band) of any tracking method known to us. From the fact that harmonic radar sets the standard for tracking of flying insects for more than two decades, [9, 21, 22, 25, 26, 30], so that new methods are always compared to it [15–17], we conclude that size is more important.

In summary, to be able to track insects over greater distances than 1000 m the tracking systems must be mobile and the tags used must be small and light to fulfil the 5% rule. There is currently no RF tracking solution that serves both since either the used RF tags are simply not small and light enough or making the receiver/tranceiver unit mobile for autonomous tracking is not possible. Therefore, it can be stated that RF tracking methods can currently not be considered as an uncompromisingly applicable solution.

In the following section we will look at visual tracking methods. Throughout the manuscript, we restrict ourselves to real-time (RT) capable systems so that they can be used as non-stationary devices in the first place. We think that stationary devices are too big a logistical challenge for the same reasons given above for RFID systems. For image-based object tracking, the frame rate and the evaluation speed (i. e. delay) is the limiting factor since a loss of the object from the field of view (FOV) usually can not be compensated. Since several years even consumer drones equipped with a vision system are capable of actively tracking and following physically untagged objects autonomously.

However, tracking small untagged objects like flying insects is not possible with a drone yet. Tracking of insects has been done for a long time, for small routes or lines of travel, with stationary devices under laboratory conditions. Nowadays, even real-time tracking of multiple small objects like flies is possible with a delay that is small enough to manipulate sensory feedback [31]. However, all such approaches have an average delay in excess of 40 ms [31–33]. This sounds small, but if we come back to the average flying speed of bumblebees (*v*), this translates to an inaccuracy of more than 28 cm. To achieve a smaller tracking error, one could slow down the flight speed for example by reducing the size of the flight arena to a relatively small one.

However, when tracking a free-flying insect with a mobile-camera, the position and orientation of the camera would have to be updated very frequently so that the small object to be tracked does not leave the FOV of the camera, as this would mean the end of tracking. In general it can be said that a high update rate of the actual insect’s position with low latency in determining its position increases the probability that an animal can be faithfully tracked. A high sampling rate can be seen as a buffer for tracking errors, since it means that more waypoints can be recorded over the same route or line of travel.

Although there are real-time (RT) visual systems to record trajectories of flying insects, they are all only capable of doing so under known, slowly changing environmental conditions, e. g. constant background. Furthermore, none of them is hard real-time capable. But the control system design of a non-stationary sensor system is made substantially easier if variability of detection lag can be ignored. This condition is met, for example, if the system is built from hard real-time components. Otherwise, the control system must have a way of dealing with sensor data of unpredictable timing. Fortunately, our system is a hard real-time (HRT) system without being restricted to slowly changing backgrounds, which makes it an ideal candidate for non-stationary tracking.

## Results and discussion

**Fig. 1:**
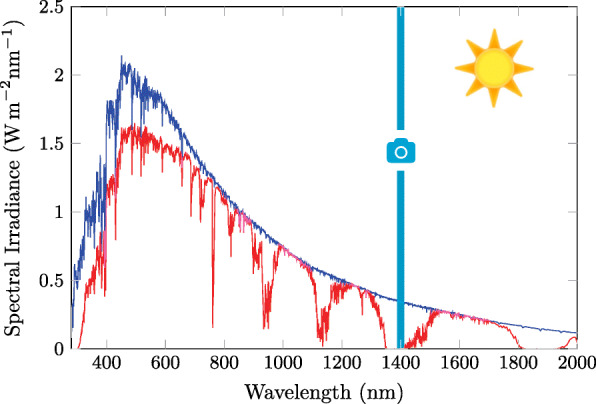
The operating window of the SWIRD detector within the solar irradiation. The difference between the terrestrial (red) solar spectral irradiance (ASTM G-173-03) and the extraterrestrial (blue) spectrum (ASTM E-490) is due to the attenuation of the signal by the atmosphere. There are areas where the electromagnetic radiation of the sun is almost completely absorbed. This is what makes tracking possible. The operating window (cyan) (i.e. the range in which the receiver () is sensitive) is given due to the full width at half maximum (25 nm and the center wavelength (1400 nm)) of the utilized band pass filter

For real-time (RT) tracking a high sampling rate is essential. Furthermore, the delay between recording and evaluation of the object’s position must always be small. To achieve a small delay complex algorithms to determine the position should be omitted, therefore a large signal to noise ratio (SNR) is essential. Thus, we were seeking for electromagnetic radiation enabling us to generate a good SNR, in other words, the signal must clearly stand out from the atmospheric and cosmic background noise — which automatically excluded the visible spectrum (Fig. 1). For the reasons mentioned above, radio waves which require an antenna on both sides — sender and receiver — are not optimal for tracking of small insects. Consequently, we were seeking for electromagnetic (EM) radiation which is not yet used for animal tracking. We were searching for a solution which would fulfill two desires, namely the reduction of size and weight of I) the transponder and II) the receiver/transceiver unit.

Based on the idea that absorption in the atmosphere of parts of the short wave infrared (SWIR) radiation promise a sound SNR, we tested if lead sulfide quantum dots (PbS QDs) which emit light with 1400 nm wavelengths are suitable to develop photoluminescence (PL) tags (Fig. 1). This would fulfill both desires due to I) an antenna free, very light transponder and II) being able to omit a sender.

For a first test we used PbS QDs in a glass vial with a round viewing window of approx. 3 mm to 4 mm diameter (for more details see material and methods). To test if the PbS QDs were suitable, we equipped a SWIR-Camera with a narrow band-pass filter with a center wavelength of 1400 nm and a full width at half maximum of 25 nm. With the prepared camera we filmed the markers under natural light conditions. We were able to detect small tagged objects easily and faced only weak levels of noise in front of various backgrounds (for more details see material and methods). The only exception were reflections of solar radiation in headlights of cars that generated medium levels of background noise. Based on the analysed videos, we concluded that the SNR was good enough to avoid a background subtraction-based algorithm in the development of the tracking method presented here. It must be mentioned that for SWIR-Sensors hot pixels and clusters of hot pixels are normal. They only need to be identified once, so they can be skipped during evaluation, which makes background subtraction unnecessary.

However, a drawback of the SWIR-cameras was the low resolution of max 640×512 pixel. Due to the combination of the small object size and the low resolution, the FOV must be small. This can be counterbalanced with higher rates of determining the object’s position i. e. high sample rates and hard real-time processing.

**Fig. 2:**
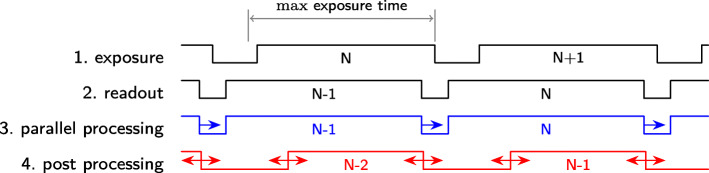
Processing of sensor data during readout vs post image processing. For our sensor, the maximum exposure time (≈10 ms) is equal to the readout time (2) if the maximum sampling rate is used. The utilized sensor was ’integrate-while-read’ capable i. e. during the readout (2) of the SWIRD sensor of the exposure (1) event *N*, exposure event *N*+1 takes place. Parallel processing (3) means that the processing starts during the readout process (2), the earliest end is the moment when readout is finished. If the latency is larger than the read out time, the end is shifted which is indicated by the blue arrow. Contrarily, post image processing (4) takes place at the earliest directly after a digital picture has been created, it can be slower or faster (indicated by the red arrow) than the readout process (2)

However, we developed a short wave infrared detection system (SWIRD) which detects the object in parallel with the readout (Fig. 2) of the matrix-detector (i.e. receiver) — thus, an extra computer is not necessary. Furthermore, we developed PL-tags necessary for tracking with an approximate weight of 12.5 mg (for more details see Methods). With the SWIRD system it was possible to record waypoints with 102 Hz. As an option the detector can function at the same time as a camera taking 102 fps (see the Availability of data and materials section for an available video). Thus, we recorded with the same device some of the flights. The exposure time was always set (slightly shorter) to ^1^ /_100_ s.

However, it was not necessary to record a video since the SWIRD receiver did the entire object detection as a stand alone device, which could communicate directly via universal asynchronous receiver-transmitter (UART) or via 1 Gbits^-1^ Ethernet with most electronic devices e. g. servo motors. Because we utilized an FPGA we could change the interface from UART to any other protocol standard (e. g. USB, CAN, I^2^C or SSI).

As long as we use the system, unintended object detection — detection of non-tagged objects — only occurred if the lens was hit by the sun directly or through total internal reflection of it, e.g. in a simple pane of glass — which can normally not happen when facing a natural environment from above. Thus, the specificity of identifying (and recording) tagged objects was 100% during the experiments.

**Fig. 3:**
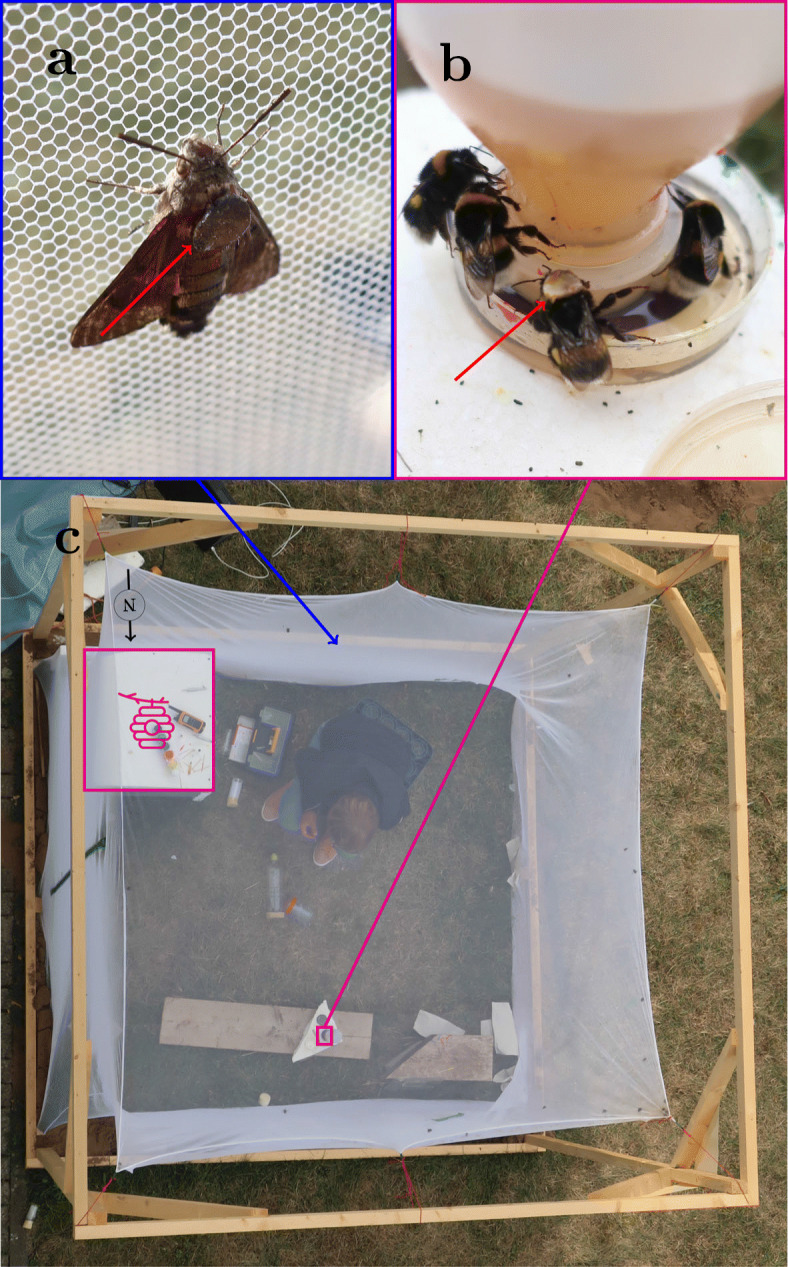
Free-flight arena with tagged individuals. In **a** and **b** the red arrow points to a PL-tag attached to a hawkmoth (a) and a bumblebee (b). In **c** the free-flight arena is shown. The picture was taken from approximately the same position where the detector was usually mounted. It represents a typical scenario during tracking with only the hive () and the feeder in a fixed position (magenta framed). Everything else could be moved freely during tracking without disturbing the recordings

With the SWIRD system we were able to track bumblebees and hawkmoths with a delay smaller than 10 ms. For the tracking of bumblebee flights a hive was moved into a cube-shaped flight arena (Fig. 3c). The bumblebees were able to fly and forage freely within this flight arena throughout the entire experimental time whereas the hawkmoths (*Macroglossum stellatarum*) were released in the flight arena only to be tracked and were removed afterwards (for more details see Methods).

**Fig. 4:**
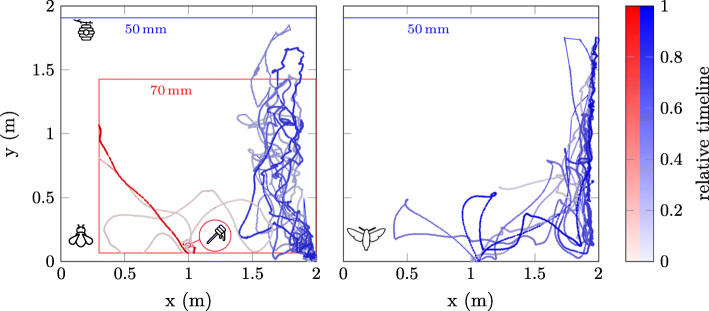
Recorded way-points with associated relative time stamps. A section (duration, 138 s, blue coloured) of an untrained bumblebees flight trajectory and a flight trajectory (duration, 164 s, red coloured) from the hive () to the feeder () and back, i.e. a foraging bout, of a trained bumblebee is shown on the left (). On the right () a section (duration, 90 s) of a hawkmoth’s flight trajectory of a long term observation flight is shown. Every mark represents one waypoint. Due to the detectors sampling rate approximately 102 waypoints were recorded per second. If no position could be determined, the last known waypoint was taken as the current one. The colour of the marks (i. e. waypoints) displays the relative timestamp. The first waypoint (with relative time stamp zero) is marked with 5% colour intensity, the last waypoint (with relative timestamp one) is marked with 100% color intensity

We recorded flights of trained (red) and untrained (blue) bumblebees (see left Fig. 4). Flights of the untrained bumblebees were recorded with a 50 mm objective whereas those of the trained individuals were recorded with a 70 mm objective. The 70 mm lens was only used for trained individuals, because we expected untrained individuals to leave its smaller FOV too often. The pros and cons of different focal lengths are discussed in more detail below. Some of the trained bumblebees that regularly visited a feeder were equipped with a tag at the start of their foraging bout (Fig. 3b). Since the individuals that were handled quickly directly departed towards the feeder, the attachment of the tag did not influence the motivation of a bumblebee to forage (see red flight left Fig. 4) as has been shown for honeybees [9]. Even though it was not necessary in this particular setup to record a direct flight after tagging, we wanted to highlight this possibility as it allows for a more unrestricted use of the method. Unlike harmonic radar antennae, our PL tags did not have to be removed after each flight, so repeated tracking was possible without additional handling of the animals. Usually, the tags remained on the animal only for one day but sometimes up to several days. Of course this is not a bumblebee-specific property, it generally applies when the attached markers last long enough. At the end of an experimental day, the animals remained marked, however, in most cases the tags got lost over night. It is not known whether animals removed them themselves or whether other animals helped. In some animals the tags also lasted longer - the longest time was four days. Thus, it would have been theoretically possible to track individual animals for hours or days, but we did not do so. We recorded individual foraging flights of trained animals and only flight sections of a few untrained animals (< 5 minutes). We did not investigate the influence of tags on behaviour. For honey bees, which are much lighter than bumblebees, it has been shown that the duration of orientation flights performed by young and inexperienced individuals was not altered significantly by harmonic radar transponders which have a comparable weight to our tags [9].

It is worth mentioning at this point that due to the flat shape of our tag the shifting centre of mass is much smaller compared to radar tags and also the aerodynamic drag is substantially less. This is of course a property of the method and independent of the marked object.

To test the tracking system on an insect species that has a high maneuverability and vastly different flight speeds within the same flight bouts [34, 35], we tracked several individuals of the hummingbird hawkmoth *Macroglossum stellatarum*. In the free-flight arena, the hawkmoths showed typical exploration and escape behaviour (flying towards the brightest side of the arena, Fig. 4). We recorded flights that were several minutes long without loosing the tracking signal for more than a few samples (Fig. 4). Since the animals could not leave the FOV when the matrix-detector was equipped with a 50 mm lens, except for a very small area at the upper edge, the system detected the hawkmoths in consecutive frames (Fig. 4). The loss of waypoints will be discussed in the following section. Overall, the three hundredfold higher temporal resolution of the tracking system compared to harmonic radar allowed us to also display intricate flight manoeuvres that occurred within single seconds.

A drawback and a property of the method is that: I) If the direct view between the detector and the insect is obstructed, or if the alignment between the PL-Tags and the detector is not optimal, tracking is not possible. Suboptimal alignment usually occurs briefly during flight maneuvers that include rotation around the roll axis, turning the tag away from the detector. Animals sitting at the wall of the flight arena were invisible to the detecor for the duration of their perch. Occlusions of the PL-tag can be caused under natural conditions by vegetation e.g. when visiting a flower. In such cases of non-detection we simply have taken the last known position as the current position. A good example is shown in left Fig. 4 since the trained bumblebee (red color) flew to the feeder where it was not perceivable for the detector for a long period of time. This is represented by the strong colour contrast of two consecutive waypoints. We assume that for many natural habitats and insect species, this simple rule of taking the last known position as the actual one is often sufficient (e.g. pollination). It is obvious that this is not the case for flights through e.g. treetops, as the FOV has to be regarded as limiting factor here, i.e. the occlusion should be smaller than the FOV of the detector. In the current version, the detector is oriented strictly in one direction. In the next version, the detector will get mounted on a gimbal, allowing for automated alignment of the FOV for object tracking. This will allow to simply centring the FOV of the detector to the last known position and holding this position until a new valid tracking point is acquired or the tracking attempt is terminated. Technically, this solution should be easy to realise. The position of a drone can be determined very accurately e.g. with the available system from Holybro (H-RTK F9P), a differential high precision GNSS positioning system (0.01 m + 1 ppm CEP, Circular Error Probable 50% of the measurements in the circle with radius *X*). Thus, together with a gimbal it is not a serious technical challenge to face.

II) There is a minimum and maximum distance between the object and the detector for which tracking is possible, depending mainly on two factors: a) The depth of field given by the lens and the aperture used. b) The maximum distance at which the amount of light emitted by the PL tag can still be detected by the system. However, we were able to record flights over the entire height of the cage. It should be noted that for the harmonic radar the covered altitude range is 3 m to 4 m [36]. Thus, we expect that for certain flights like foraging flights this limitation is rather unproblematic [37], and might rather pose problems for flights with a high variance in altitude. Using the detector as a non stationary airworthy device, distance detection, i.e. a three-dimensional determination of the position of the object to be tracked, would be an appropriate solution to overcome this limitation, e. g. with stereoscopic vision or implementing contrast detection.

Despite the fact that we were able to record animals over the entire height of the flight arena, animals could not always be tracked: I) Because the FOV of the detector (for both utilized lenses) was not covering the whole flight arena. The likelihood of an animal leaving the FOV depends on the size of the FOV, thus it depends on the focal length of the lens. Consequently, insects left the FOV more often when we used the 70 mm lens than with the 50 mm lens (see left Fig. 4). II) Because of tracking errors induced through its small size, a smaller FOV counteracts for it since a tag of the same size is illuminating a larger area on the sensor. This is important if the tag is smaller than four times the area covered by a pixel or if the tag is aligned in such a way that the maximum visual surface of the tag is smaller than this for a given FOV of the lens with the larger focal length. Up to this visual tag size the maximum signal value (if the strongest signal is perceived without clipping) perceived by the detector varies depending on the position of the tag.

As mentioned above, utilizing a large focal length leads to many advantages besides the disadvantage of a smaller FOV. We believe that the disadvantages of a smaller FOV of the 70 mm objective compared to the 50 mm objective can be compensated by mounting the detector on a self-tracking gimbal. In this case the number of records that can be missed is crucial because the more records are allowed to be lost before the FOV is left by the insect the more likely tracking will work. In the setup used, with the 70 mm lens, approx. 85 ms elapse when an insect moves along the shortest path with 8 ms^-1^ from the centre to the edge of the field of view. During this time eight samples can be taken by the detector which seems to be a reasonable buffer for autonomous gimbal tracking (see red flight trajectory, left Fig. 4).

With our method we were able to track bumblebees and hawkmoths. Since the size of the tag can be reduced, we are confident that tracking of smaller flying insects can also be achieved. We expect that even without any technical progress, tags with a radius of 3.6 mm and a weight of 6.5 mg can be tracked with the 70 mm lens, as it is only scaling (if all other optical properties of the lenses are negligible), in a comparable quality as recordings with the 5 mm tags and the 50 mm lens.

Due to the fact that the utilized tags were at least three f-stops darker compared to PBS QDs in toluene (10 mg mL^-1^), one can assume that with technical progress — increasing the PL-intensity of the tags — even smaller insects can be tracked. Since the passivation method described by Bederak et al. [38] is most likely superior to ours, it should be possible to build tags with grater PL luminosity by using this method.

However, at this stage of development the SWIRD system is a stationary device but it is already designed to be used as a non stationary tracking device. Thus, tracking unrestrained free-flights even of small insects over distances greater than 1000 m is within reach.

## Conclusions

In this study we present a new method to track flying insects under natural-like conditions. We believe that the method we present is the foundation for a technological leap that will allow to record insect routes of travel over long distances in great detail. This can be an essential contribution to address questions with a very thin knowledge base so far, e. g. navigation and orientation of non central place foragers. The SWIRD system is already designed to be used as a non stationary tracking device. It can detect PL-tagged objects and can pass the specified position to other electronic devices. Furthermore, the device weighs less than 380 g, including a 70 mm focal lens, which is light enough to be attached to a gimbal and mounted on a relatively small multicopter (drones under 4 kg take off weight). Therefore, according to the European Union Aviation Safety Agency (EASA), the technique could be used even in urban areas.

## Methods

### Preliminary tests

The basic concept of the idea of marking objects using PbS QDs with a PL emission peak at 1400 nm (CAS Number: 1314-87-0 by Strem Chemicals, Inc.), was investigated in a pre-experiment. Here we used a commercial camera (Bobcat 640 from Xenics) and a 1400 nm band-pass filter, that was placed in front of the lens. The marker was simulated by a small ampulla filled with PbS QSs and sealed with light-tight tape, that had a small hole of 3 mm to 4 mm diameter. The image delivered by the camera showed a black screen with a small white dot, representing the opening in the ampulla. In further steps, we chose different natural backgrounds (e. g. wet and dry meadows, wet and dry leaves) and urban backgrounds (e. g. tarred and paved roads, cars) to see, if the appearance of the image was affected. These tests were carried out in the live view mode of the camera.

### Technical basis

A SWIRD system consist of two main components, i. e. the marker (PL-tag) and the detector (the receiver).

#### PL-tag

The prototype markers were made using a round papercutting (80 g m^-2^, white paper), made with a common hole punch, with a diameter of 5 mm (Fig. 3a and b).

It was coated with lead sulfide quantum dots (PbS QDs) with a PL emission peak at 1.4 *μ*m (CAS Number: 1314-87-0 by Strem Chemicals, Inc.) which is very close to the absorbance peak of water (1.45 *μ*m, water vapor (1.38 *μ*m) and carbon dioxide (1.4 *μ*m) [39–41], which leads to almost zero electromagnetic radiation in this region of solar radiation on the surface of the earth (Fig. 1). Since PbS QDs are quickly oxidized and lose their PL properties under normal atmosphere, a passivation layer of UV cured transparent glue (Vitralit 7041 by Panacol) was used to cover them.

Due to the susceptibility of the tags to oxygen, we tested them for functionality with the detector on the day of the experiment. Thus, the passivation of the tags prevents the animals from contacting PbS. Furthermore, if the passivation layer is damaged, the lose their luminescence within a very short time (probably less than one minute). The final mass of each marker was approximately 12.5 mg.

#### Detector

The detector of the SWIRD system is sensitive to 1400 nm electromagnetic (EM) radiation. Furthermore, the SWIRD system is able to determine the position of the marker, on the pixel array of the detector, i. e. the direction of the marker. The exact distance between detector and tag was unknown and was assumed to be seven meters — assuming that the tags were halfway up the arena at the time of tracking. Under this assumption, the maximum deviation between tracked position and actual position (in x and y) was less than 11.2 cm and would occur, if the animal was at the height of the ceiling or floor. The detector was composed of three main modules, i. e. the optics, the sensor and the programmable logic unit.

The detector was equipped with a Pentax lens mount, that allowed to choose different standard Pentax lenses. For our experiments two lenses were used: a Sigma f/1.4 50 mm F1.4 Ex DG HSM lens with a weight of 555 g and a SMC Pentax DA 70mm F2.4 lens with a weight of 136 g. The Pentax system was used, since a simple mechanical modulation of the aperture could be realized by an external micro servo. Behind the lens a band-pass filter with a central wavelength of 1400 nm was installed. We chose a filter by Edmund Optics with an optical density of 4, a full width at half maximum of 25 nm and a diameter of 25 mm. Light passing the filter then finally reached the central component of the detector, a two dimensional Indium-Gallium-Arsenid (InGaAs) focal plain array with 640×512 elements from ADANTA GmbH. Each element had a dimension of 25 *μ*m by 25 *μ*m. It was sensitive for EM radiation with a wavelength in the range from 900 nm to 1700 nm and could be read out 102 times per second. The sensor was controlled by a digital interface, but the sensor output was analog. The analog output was then digitized by an analog-front-end (AFE, VSP5610 by Texas Instruments Inc.), creating an 8 bit digital representation of the EM intensity for each sensor element. The data rate of the AFE output is given by 640 B×512 B×102 s^−1^≈33,4 MB/s. All further information necessary for this step was retrieved from the manufacturers’ data sheets (for more details see Availability of Data and Materials).

This data stream then needed to be handled by a programmable device. Our choice for this task was a Field Programmable Gate Array (FPGA, Spartan 6 xc6slx45 from Xilinx). An FPGA has some advantages compared to a Central Processing Unit (CPU) based architecture, which would be an option, too. An FPGA can handle data in parallel and the processing is hard-coupled to the system clock, i. e. the execution time for every process is constant. This leads to a constant processing time for every measurement (hard real-time processing), which is important for a following control, as depicted before. The only cause for jittering of the processing time is the central clock jitter, which is in the range of parts per million, and thus negligible.

The main task within the FPGA was the extraction of the position of the PbS marker, and thus the marked animal, from the (analogue) image taken by the sensor. The core algorithms were written in VHDL (VHDL-2008), and are freely available (see Availability of data and materials section). Since the only object visible in each (analogue) image was the marker, the position detection could be done pixelwise by simple mathematical operations without utilising background subtraction, i. e. not a whole image was necessary. The calculation was done in parallel to the read out of every row (*i*) of the 256 grey level 512×640 matrix detector. We determined for every pixel (*a*_*i*,*j*_) in the natural order if its value was lager than a given threshold (T ∈{0,1,…,255}). If the value was larger than T, this pixel position was stored (*i*,*j*) and the previous pixel position was overwritten. At the end of the image at position (*a*_512,640_) the actual detected position was returned. If during a readout process no object was detected, in other words no pixel had a value greater than *T*, e.g. due to occlusion or rotation of the body axis of the insect to be observed, the value (0,0) was returned. This detection process is a simple and straight forward approach which could easily be implemented in an FPGA. Since the position detection was applied in parallel to the readout process of the sensor, the position of the marker was known at the moment, when the last pixel has been read from the sensor. This is the minimal possible delay between exposure and the delivering of the result, i. e. the position of the marker (Fig. 2). The reduction of information from the whole image to the position of the marker in the image led to an extraordinary reduction of the data volume produced by the detector. Finally, only 0.4 kB s^-1^ of data were output by the device.

Implemented for the information output are now UART and a 1 Gbits^-1^ Ethernet interface. The latter can also be used to send the raw image at the same time, which is helpful for debugging. Thus, our matrix detector can also be used as a camera. The final mass of the detector without the lenses was only 244 g. Mounting the 70mm F2.4 lens we gained a full functional detector with a total mass of 380 g.

### Experimental setup

The flight arena was constructed using wooden slats, that held a 2 m×2 m×2 m mosquito net. The white net was specified as having 220 holes per square inch (see Fig. 3c). Due to the Corona pandemic, we could not predict exactly when and where we would be able to conduct the trials. Thus, by using a flight arena, we did not need any permits: I) to set up the bumblebee hive II) to release laboratory animals (hawkmoths). The arena was placed on the western side of a building onto a meadow which had not been altered in any way and therefore represented a section of a natural habitat. The detector was mounted above the net at a height of 8 m, from the same height the image of Fig. 3c was taken (deviation from the detector position approximately 1.5 m in eastern direction). The diameter of the marker was defined by this setup - namely by the field of view (FOV) of the detector and by the distance between marker and detector. Since the resolution of the sensor was 640×512, one element of the sensor covered an area of ≈ 3,9 mm by 3,9 mm. We chose a marker diameter of 5 mm, since we obtained a signal quality good enough to be detected. Changing the setup, e. g. by decreasing the arena ground area would allow for smaller a marker size.

#### Bumblebees

A bumblebee hive was positioned in one corner of the flight arena (Fig. 3c). We trained a group of bumblebees to a feeder location in the middle on the opposite side of the arena (Fig. 3c). This feeder was filled with APIINVERT (Südzucker AG, Mannheim, Germany) as a food source and foragers visited it regularly once the training was completed. All regular foragers were marked with color on the abdomen to be easily identified as potential experimental animals. For the attachment of the PL-tag an experimental animal was caught at the entrance of the hive shortly after it departed to fly towards the feeder. We used nontoxic shellac (“Opalith Zeichenleim” included in the following set of “Opalith Zeichenplättchen”, EAN: 4060932575218) to glue the PL-tag to the thorax (see Fig. 3b) and released it afterwards at the same location as quick as possible to record its flight towards the feeder.

#### Hawkmoths

Hummingbird hawkmoths were taken from populations that were reared under laboratory lighting conditions. They were immobilised under a net which left their thorax free, to remove their scales and glue a PL-tag on (see Fig. 3a) using a multi-purpose impact instant contact adhesive (EVO-STIK, Bostik Ltd, Stafford, UK). The animals were kept in a dark container to let the glue dry for at least 15 min. before being adapted to the outdoor light conditions for a minimum of 15 min. They were then released into the outdoor flight arena to be tracked.

## Data Availability

The camera/detector is a unique material that cannot be made widely available but the information needed to construct an identical or similar device can be found in the methods and on GitLab (https://gitlab.com/airworthyd/swird). The core algorithms were written in VHDL (VHDL-2008). The GUI Software was developed with Lazarus (Lazarus 2.0.10) using the Free Pascal (FPC 3.2.0) compiler, and all source files are available on GitLab (https://gitlab.com/airworthyd/swird) The complete datasheet for the AFE is available from Texas Instruments Inc. The complete datasheet for the sensor is available from Andanta GmbH.

## Abbreviations

AFE: Analog front end
EM: Electromagnetic
FOV: Field of view
FPGA: Field programmable gate array
HRT: Hard real-time
PbS: Lead sulphide
PL: Photoluminescence
QD: Quantum dots
RADAR: Radio detection and ranging
RF: Radio frequency
RFID: Radio frequency identification
RT: Real-time
SNR: Signal to noise ratio
SWIRD: Short wave infrared detection
SWIR: Short wave infrared
UART: Universal asynchronous receiver-transmitter
